# Increased neutrophil–lymphocyte ratio predicts recurrence in patients with well-differentiated pancreatic neuroendocrine neoplasm based on the 2017 World Health Organization classification

**DOI:** 10.1186/s12893-021-01178-3

**Published:** 2021-03-31

**Authors:** Takayuki Miura, Hideo Ohtsuka, Takeshi Aoki, Shuichi Aoki, Tatsuo Hata, Tatsuyuki Takadate, Shimpei Maeda, Kyohei Ariake, Kei Kawaguchi, Kunihiro Masuda, Masaharu Ishida, Masamichi Mizuma, Kei Nakagawa, Takanori Morikawa, Fumiyoshi Fujishima, Takashi Kamei, Hironobu Sasano, Michiaki Unno

**Affiliations:** 1 Department of Surgery, Tohoku University Graduate School of Medicine, 1-1 Seiryo-machi, Aoba-ku, Sendai, Miyagi 980-8575 Japan; 2 Department of Pathology, Tohoku University Graduate School of Medicine, Sendai, Japan

**Keywords:** Neutrophil–lymphocyte ratio (NLR), Pancreatic neuroendocrine neoplasm (PanNEN), Systemic immune-inflammatory marker

## Abstract

**Background:**

The prognostic values of inflammation-based markers in well-differentiated pancreatic neuroendocrine neoplasms, diagnosed according to the new 2017 World Health Organization classification, have remained unclear. Therefore, we assessed the ability to predict the recurrence of such markers after curative resection in patients with these neoplasms.

**Methods:**

Circulating/systemic neutrophil–lymphocyte, monocyte–lymphocyte, platelet–lymphocyte, and platelet–white cell ratios were evaluated in 120 patients who underwent curative resection for well-differentiated pancreatic neuroendocrine neoplasms without synchronous distant metastasis between 2001 and 2018. Recurrence-free-survival and overall survival were compared using Kaplan–Meier analysis and log-rank tests. Univariate or multivariate analyses, using a Cox proportional hazards model, were used to calculate hazard ratios with 95% confidence intervals.

**Results:**

Univariate analysis demonstrated that preoperative neutrophil–lymphocyte ratio, tumor size, European Neuroendocrine Tumor Society TMN classification, 2017 World Health Organization classification, and venous invasion were associated with recurrence. The optimal preoperative neutrophil–lymphocyte ratio cut-off value was 2.62, based on receiver operating characteristic curve analysis. In multivariate analysis, a higher preoperative neutrophil–lymphocyte ratio (HR = 3.49 95% CI 1.05–11.7; P = 0.042) and 2017 World Health Organization classification (HR = 8.81, 95% CI 1.46–168.2; P = 0.015) were independent recurrence predictors.

**Conclusions:**

The circulating/systemic neutrophil–lymphocyte ratio is a useful and convenient preoperative prognostic marker of recurrence in patients with well-differentiated pancreatic neuroendocrine neoplasm based on the 2017 World Health Organization classification.

**Supplementary Information:**

The online version contains supplementary material available at 10.1186/s12893-021-01178-3.

## Background

Pancreatic neuroendocrine neoplasm (PanNEN) is a biologically heterogeneous and relatively rare malignancy, with an incidence rate of approximately 5 cases per 1 million person-years, which accounts for 1–2% of primary pancreatic neoplasms [1]. In recent years, the incidence of PanNEN detected clinically has significantly increased because of the advances in imaging modalities during the past few decades [2]. When the disease is clinically detected before it becomes symptomatic, the lesions are typically localized, increasing the possibility of curative resection and improving prognosis [3]. Although surgical resection is currently the only curative treatment for PanNEN [4], recurrence could occur at intervals, and therefore, reoperation for recurrent lesions may occasionally be required. Reoperation for distant metastases can lead to excellent long-term survival [5]. Even if unresectable metastases occur, novel targeted drugs, such as the multiple tyrosine kinase inhibitor sunitinib and the mTOR inhibitor everolimus, have been approved and registered for antiproliferative therapy for well-differentiated PanNEN [6, 7]. Therefore, it is essential to identify recurrence earlier. For this reason, indicators that could predict recurrence after surgery are required for the optimal management of PanNEN.

Several studies, however, have demonstrated that tumorigenesis and clinical manifestations of well-differentiated PanNEN are distinctively different from poorly differentiated PanNEN (neuroendocrine carcinoma; NEC), and thus, the determinants of treatment should be considered separately [8, 9]. The prognosis in NEC is poor [10], and the National Comprehensive Cancer Network (NCCN) guidelines recommend platinum-based systemic chemotherapy for patients with NEC [6]. For this reason, the 2017 World Health Organization (WHO) introduced significant changes to the classification of PanNEN. Of note, a new category of well-differentiated neoplasms, neuroendocrine tumors G3 (NET-G3), was introduced, and these are distinct from poorly differentiated NEC-G3 [11, 12].

Recently, systemic immune-inflammatory markers have been reported as factors that influence the outcomes of treatments, such as surgery, and the efficacy of chemotherapy in patients with various types of malignancies [13–18]. However, the ability of systemic immune-inflammatory markers to predict prognosis in patients with sole, well-differentiated PanNEN, based on the 2017 WHO classification [11], other than NEC or synchronous distant metastasis, has remained unknown.

In this study, we sought to evaluate whether systemic immune-inflammatory markers can be preoperative prognostic factors for predicting recurrence and overall survival after curative resection in patients with well-differentiated PanNEN based on the new 2017 WHO classification.

## Materials and methods

### Patients

We analyzed 132 consecutive cases who underwent surgery for primary, histologically confirmed PanNEN at the Department of Surgery, Tohoku University Hospital, between 2001 and 2018. Eight patients with synchronous hepatic metastasis during the surgery, two patients with NEC, one patient not suitable for curative resection, and one patient with an active infection at blood sampling were excluded from the study. Finally, 120 patients with well-differentiated PanNEN were enrolled in this study. Patient characteristics (age, sex), perioperative factors (serum albumin, hormonal secretion, tumor location, clinical stage), pathological findings (2017 WHO classification, tumor size, lymph node metastasis, surgical margin status, lymphovascular invasion), and prognosis were investigated retrospectively. Histopathological findings were assessed by experienced pathologists (FF, and HS). For all the patients, visual assessment ‘‘eyeballing calculation’’ was performed to assess Ki-67 index. TNM staging was adopted according to the European Neuroendocrine Tumor Society (ENETS) classification [19], and the new 2017 WHO classification of NET by the gastro-entero-pancreatic (GEP) system was used for histopathological classification [11]. Peripheral blood routine tests were performed within 14 days before surgery, according to our internal institutional policy. The serum neutrophil–lymphocyte ratio (NLR) was calculated as the number of neutrophils divided by the number of lymphocytes. The monocyte–lymphocyte ratio (MLR), platelet–lymphocyte ratio (PLR), and platelet–white cell ratio (PWR) were calculated in the same manner.

### Clinical follow-up

Postoperative follow-up evaluation included physical examinations, laboratory tests, and enhanced computed tomography (chest and abdominal cavity), once every 6 months. There were no patients who received neoadjuvant or adjuvant therapies in this cohort. Treatment after recurrence was determined by the available evidence at the time of surgery and based on the patient’s condition.

### Statistical analysis and software

Recurrence-free-survival (RFS) and overall survival (OS) were calculated from the date of surgery to the date of recurrence, the date of death from any cause, or the date of last follow-up. To determine the appropriate cut-off values, we used receiver operating characteristic (ROC) curves and determined the area under the curve (AUC). Differences between groups were determined using t-tests in the case of normally distributed variables or by the Wilcoxon rank-sum test in the case of abnormally distributed variables for examining differences in continuous variable distributions, and Pearson’s chi-square tests for categorical variables. RFS probabilities were compared for various categories of interest using the Kaplan–Meier method with the log-rank test.

Prognostic factors were assessed with univariate and multivariate analyses, using Cox’s proportional hazards model. Hazard ratios (HR) with 95% confidence intervals (CIs) were calculated. P < 0.05 was considered to indicate statistical significance.

All statistical analyses were performed using the JMP Pro 14.2.0 statistical software (SAS Institute Inc., Cary, NC, USA) and GraphPad Prism Version 8.4.2 (GraphPad Software, San Diego, CA, USA).

### Ethics approval

This study was approved by the ethics committee of the Tohoku University Hospital (Approval No. 2020-1-322). It was performed in adherence to the tenets of the Declaration of Helsinki and its later amendments. The need to obtain written informed consent was waived due to the retrospective nature of the study.

## Results

### Characteristics of patients with resected well-differentiated PanNEN

The demographic and clinicopathological features of the 120 patients who underwent curative resection of PanNEN are shown in Table 1. The median age was 60 years (range 12–88 years), and the median follow-up period in all patients was 64 months (range, 6–185 months). There were no perioperative deaths. The pathological findings (based on the 2017 WHO classification) were NET-G1 in 73 patients, NET-G2 in 45, and NET-G3 in 2. The median tumor size was 14.5 mm (range 4–168 mm). Pathology investigations confirmed lymph node metastasis in 18 patients (15.0%).

**Table 1:** Clinicopathological characteristics of 120 patients with well-differentiated PanNEN

Patient characteristics	n = 120	%
Age, median(range)	60 (12‒88)	
Sex		
Male	49	40.8
Female	71	59.2
NLR, median (range)	1.93 (0.44‒5.32)	
MLR, median (range)	0.23 (0.11‒0.53)	
PLR, median (range)	145.2 (42.5‒328.8)	
PWR, median (range)	43.7 (11.9‒120)	
Albumin(g/L), median (range)	41.0 (28‒49)	
Tumor size (mm), median (range)	14.5 (4‒168)	
Operative procedures		
PD	38	31.7
DP	58	48.3
TP	2	1.7
Partial resection	22	18.3
Surgical approach		
Open	78	65.0
Laparoscopy	42	35.0
Surgical margin status		
R0	116	96.7
R1	4	3.3
Tumor location		
Head	47	39.2
Body/tail	68	56.7
Multiple	5	4.2
Ki-67 (%), median (range)	1.83 (0.02‒28)	
Clinical stage		
I	74	61.7
II	27	23.3
III	19	15.0
2017 WHO classification		
G1	73	60.8
G2	45	37.5
G3	2	1.7
Hormonal function		
No	70	58.3
Yes	50	41.7
Lymph node metastasis		
No	102	85.0
Yes	18	15.0
Lymphatic invasion		
No	105	87.5
Yes	15	12.5
Venous invasion		
No	91	75.8
Yes	29	24.2

Data are expressed as the median (range) or as absolute number

*NLR* neutrophil–lymphocyte, *MLR* monocyte–lymphocyte ratio, *PLR* platelet–lymphocyte, *PWR* platelet–white blood cell ratio, *PD* pancreaticoduodenectomy, *DP* distal pancreatectomy, *TP* total pancreatectomy

### Clinicopathological features associated with recurrence and NLR

**Fig. 1:**
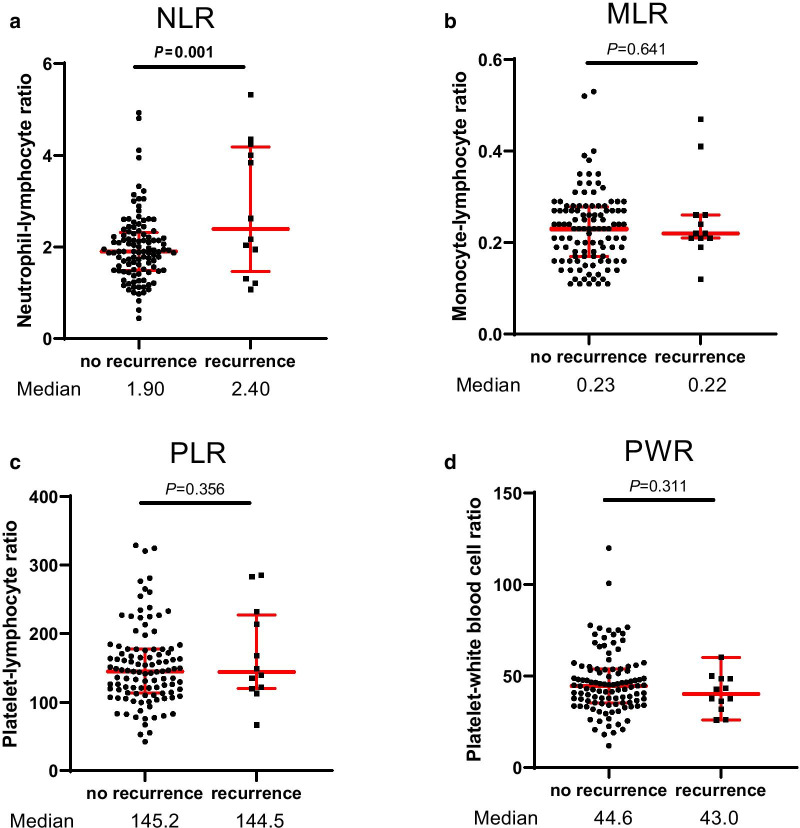
Distribution of the inflammation-based markers in PanNENs. The NLR was significantly higher in patients with recurrence than in those without recurrence, while the MLR, PLR, and PWR were not statistically different between those with and those without recurrence

Postoperative recurrences were observed in 12 cases (10%). The sites of recurrence were in the liver in 10 patients, the para-aortic lymph node in 1, and the lung in 1. The 5- and 10-year RFS rates for the entire cohort were 92.0% and 78.7%, respectively. Three patients died due to PanNEN, 10 patients died due to other diseases, and the remaining 107 patients were alive at the end of the surveillance period. Thus, the 5- and 10-year disease-specific survival rates were 100% and 92.5%, respectively. The NLR was significantly higher in patients with recurrence than in those without recurrence (median NLR: 2.40 vs 1.90, P = 0.001), while the MLR, PLR, and PWR were not statistically significantly different between those with and those without recurrence (Fig. 1).

An ROC curve was used to determine the cut-off value associated with postoperative recurrence. Each cut-off value of NLR and tumor size was defined as the highest log-rank statistic of any threshold. The optimal cut-off values for preoperative NLR and tumor size were 2.62 mm and 25 mm, respectively (Additional file 1: Fig. S1.). The Ki-67 index was statistically higher in patients with high NLR (≥ 2.62) than in patients with low NLR (< 2.62) (mean: 5.46 vs 3.14, P = 0.042). In contrast, age, sex, albumin, surgical margin status, clinical stage, 2017 WHO classification, tumor functionality, tumor size, tumor location, lymph node metastasis, and lymphovascular invasion were not associated with NLR status (Table 2). The recurrence rate was 33.3% and 31.0% in 18 patients with a high NLR (≥ 2.62) and 29 patients with larger tumors (≥ 25 mm), respectively.

**Table 2:** Relationship between NLR and clinicopathological characteristics (n = 120)

	LNR < 2.62 (n = 102)	LNR ≥ 2.62 (n = 18)	*P*-value
Age (years)	57.7 ± 16.0	59.5 ± 14.7	0.763
Sex			
Female	59	12	0.483
Male	43	6	
Albumin (g/L)	3.98 ± 0.40	4.01 ± 0.41	0.915
Tumor size (mm)	19.0 ± 19.4	22.6 ± 15.1	0.194
Surgical margin status			
R0	99	17	0.569
R1	3	1	
Tumor location			
Head	39	8	0.595
Body/tail	58	10	
Multiple	5	0	
Ki-67	3.14 ± 3.83	5.46 ± 6.97	**0.042**
Clinical stage			
I	64	10	0.714
II	23	4	
III	25	4	
2017 WHO classification			
G1	64	9	0.361
G2	37	8	
G3	1	1	
Hormonal function			
No	56	14	0.070
Yes	46	4	
Lymph node metastasis			
No	88	14	0.352
Yes	14	4	
Lymphatic invasion			
No	88	17	0.334
Yes	14	1	
Venous invasion			
No	77	14	0.834
Yes	25	4	

Results are expressed as mean ± SD or as absolute number

### Comparison of clinical variables in relationship to RFS after curative resection

**Fig. 2:**
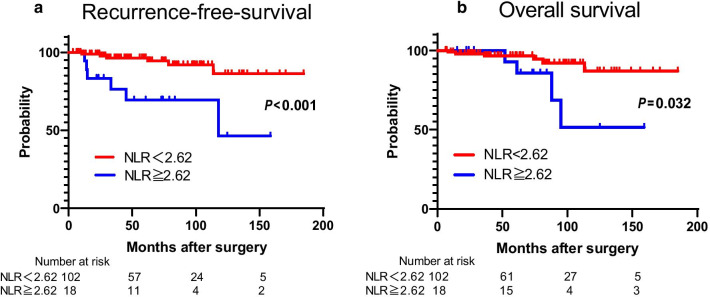
Recurrence-free-survival and overall survival for PanNENs stratified by NLR. A higher NLR showed a significant correlation with shorter RFS (median RFS duration, 117.8 months, P < 0.001) (**a**) and poor OS (median OS duration, 95.2 months, P = 0.032) after curative resection (**b**)

The results of the univariate and multivariate analyses for each of the clinicopathological variables are shown in Table 3. According to univariate analysis, the recurrence risk was about six times higher in patients with a high NLR than in those with a low NLR (95% CI 1.81–18.5, P = 0.004). Additionally, the TMN clinical-stage, 2017 WHO classification G2/3, tumor size, and venous invasion were also significantly predictive factors for recurrence (P < 0.05 for all). In contrast, age, sex, albumin, surgical margin status, hormonal function, tumor location, lymph node metastasis, and lymphatic invasion were not significant predictors of recurrence. Moreover, in multivariate analysis, higher NLR (HR = 3.49, 95% CI 1.05–11.7, P = 0.042) and 2017 WHO classification G2/3 (HR = 8.81, 95% CI 1.46–168.2, P = 0.015) were independent predictive factors for recurrence. A higher NLR showed a significant correlation with shorter RFS (median RFS duration, 117.8 months, P < 0.001) (Fig. 2a) and poor OS (median OS duration, 95.2 months, P = 0.032) after curative resection (Fig. 2b).

**Table 3:** Prognostic factors for recurrence-free-survival in 120 patients with well-differentiated PanNEN

Independent factor	Univariate analysis			Multivariate analysis		
	Hazard ratio	95% CI	*P*‒value	Hazard ratio	95% CI	*P*‒value
Age (years)			0.101			
< 60	Reference					
≥ 60	0.36	0.08‒1.21				
Sex			0.09			
Female	Reference					
Male	2.66	0.84‒9.05				
NLR			**0.004**			**0.042**
< 2.62	Reference			Reference		
≥ 2.62	5.78	1.81‒18.5		3.49	1.05‒11.7	
Albumin (g/L)			0.829			
< 35	Reference					
≥ 35	0.79	0.15‒14.5				
Tumor size (mm)			** < 0.001**			0.052
< 25	Reference			Reference		
≥ 25	10.2	3.05‒46.2		5.30	0.98‒81.5	
Surgical margin status			0.337			
R0	Reference					
R1	2.74	0.35–21.5				
Tumor location			0.619			
Head	Reference					
Body/tail	0.95	0.28‒2.99				
Multiple	NA	NA				
Clinical stage			**0.001**			0.736
I	Reference			Reference		
II/III	8.12	2.13‒52.9		1.19	0.06‒13.6	
2017 WHO classification			** < 0.001**			**0.015**
G1	Reference			Reference		
G2/G3	15.6	3.02‒285.6		8.81	1.46‒168.2	
Hormonal function			0.151			
No	Reference					
Yes	2.46	0.73‒11.1				
Lymph node metastasis			0.063			
No	Reference					
Yes	3.49	0.93‒11.1				
Lymphatic invasion			0.150			
No	Reference					
Yes	2.89	0.64‒9.77				
Venous invasion			**0.022**			0.356
No	Reference			Reference		
Yes	3.96	1.23‒12.7		1.17	0.29‒4.49	

Variables associated with RFS according to the Cox proportional hazards regression model

*RFS* Recurrence-free-survival, *NLR* neutrophil–lymphocyte ratio, NA not available

P-value < 0.05 marked in bold font shows statistical significance

### Subgroup analyses of the hormonal function associated with the NLR

**Fig. 3:**
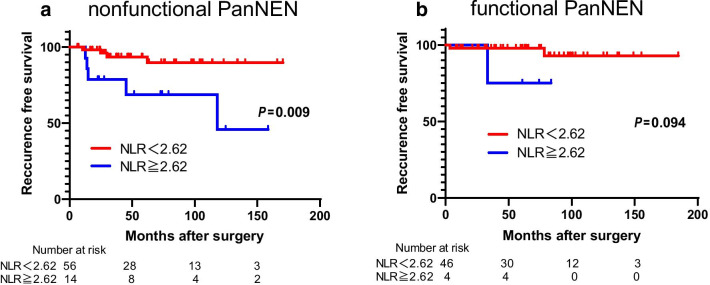
Recurrence-free-survival for nonfunctional and functional PanNENs stratified by NLR. A higher NLR was significantly associated with a shorter RFS in nonfunctional PanNEN (**a**). Contrary to nonfunctional PanNEN, NLR was not associated with RFS in functional PanNEN (**b**)

We then focused on the usefulness of the NLR for the classification of functional and nonfunctional PanNEN. We confirmed a strong association between NLR and RFS, especially in nonfunctional PanNEN (HR 4.95; 95% CI 1.430–20.1; P = 0.002) (Table 4). Additionally, a higher NLR was significantly associated with a shorter RFS in nonfunctional PanNEN (P = 0.009) (Fig. 3a). Contrary to nonfunctional PanNEN, NLR was not associated with RFS in functional PanNEN (P = 0.094) (Fig. 3b).

**Table 4:** Subgroup analysis for recurrence-free-survival according to neutrophil–lymphocyte ratio

NLR	n (%)	RFS		
		Hazard ratio	95% CI	*P*‒value
Nonfunctional PanNEN				
< 2.62	56 (80)	Reference		**0.002**
≥ 2.62	14 (20)	4.95	1.30‒20.1	
Functional PanNEN				
< 2.62	47 (94)	Reference		0.198
≥ 2.62	3 (6)	6.18	0.28‒66.4	

P-value < 0.05 marked in bold font shows statistical significance

## Discussion

The current study demonstrated that elevated preoperative NLR and 2017 WHO classification independently predicted recurrence in patients with well-differentiated PanNEN after curative surgery. No previous studies have demonstrated that increased NLR serves as an independent prognostic factor in patients with nonmetastatic well-differentiated PanNEN, as defined by the 2017 WHO classification. This may be of potential clinical benefit in these patients. Furthermore, we observed that elevated preoperative NLR was predictive of a significantly shorter RFS in nonfunctional PanNEN patients.

Previously, PanNEN with lymph node metastasis, a higher Ki-67 index, and a higher 2010 WHO grade were reported to be associated with a significantly higher risk of recurrence [20, 21]. In contrast, a large international cohort study showed that the ENETs TNM classification was superior to the Union for International Cancer Control/American Joint Committee on Cancer/WHO staging system and could more accurately predict the clinical outcome of patients [22]. We revealed that the ENETs TNM classification was related to RFS in univariate analysis but not in multivariate analysis. A possible explanation for this finding is that we assessed the patients with curatively resected PanNEN and excluded metastatic stage IV patients in our present study. Clinically, the preoperative Ki-67 index obtained by fine-needle aspiration biopsy is less accurate due to intra-tumoral heterogeneity [23], which highlights the requirement of preoperative non-invasive prognostic indicators, such as inflammation-based markers. Preoperative precise assessment of recurrence risk of the patients allows clinically more relevant selection of an optimal surgical strategy, such as enucleation or further lymph node dissection.

In terms of systemic inflammation-based markers in PanNEN, preoperative NLR and PLR have been reported to be useful for predicting lymph node metastasis or recurrence [24–28]. However, these studies included a moderate number of patients with distant metastatic stage IV or poorly differentiated PanNEN (NEC), as defined as 2010 WHO NET-G3. Generally, poorly differentiated PanNEN has substantial distant metastases and a distinctly poor prognosis [29]. The 2010 WHO classification of NET-G3 included both well-differentiated and poorly differentiated PanNEN, resulting in a morphologically and biologically heterogeneous population [30]. Consequently, the 2017 WHO classification of NET-G3 was recategorized as only well-differentiated PanNEN, distinctively different from NEC. Indeed, the median RFS (6.7 months) and median OS (15.3 months) of surgically resected NEC were markedly shorter than in well-differentiated PanNEN in our institute (Additional file 1: Fig. S2.). Furthermore, the value of NLR for NEC was significantly higher than that in patients with well-differentiated PanNEN (Additional file 1: Fig. S3.). In our present study, we assessed the efficacy of NLR to predict recurrence in well-differentiated PanNEN, other than NEC or distant metastasis, based on the 2017 WHO classification. Hence, more prolonged RFS and OS were detected in our present study than in the previously reported ones [24–28].

NLR was recently reported to be associated with tumor progression in several human malignancies [14–18]. In addition, NLR could serve as a predictive marker in patients with not only PanNEN but also gastrointestinal NEN [27]. We previously reported that the NLR was a useful diagnostic marker for predicting intraductal papillary mucinous neoplasm with high-grade dysplasia/invasive carcinoma to differentiate low-grade dysplasia [31]. Previous studies reported that a high NLR was significantly consistent with accumulation of tumor infiltrating CD66b neutrophils or CD163^+^ macrophages in patients with PanNEN and pancreatic cancer, which results in poor RFS and OS [28, 32]. In general, neutrophils are markers of acute inflammation and could possibly promote tumor development and progression by providing an adequate tumor microenvironment via the production of cytokines and chemokines [33]. In addition, an increased number of lymphocytes play a crucial role in the host’s anticancer immune response; thus, lymphocytosis is generally associated with a better prognosis and a more favorable response to chemotherapy or radiation therapy in a variety of cancers [34]. Therefore, in cancer patients, peripheral blood neutrophilia and lymphopenia may reflect a weak anticancer reaction and worse clinical outcomes [35].

Regardless of the histological findings, there are hormonally functional and nonfunctional phenotypes in PanNEN. According to an epidemiological survey, the number of PanNEN patients has increased rapidly. In particular, hormonally nonfunctional PanNEN was most prevalent and increased significantly [36, 37]. To the best of our knowledge, no previous studies have demonstrated possible roles of NLR as a prognostic factor for RFS in distinct categories of nonfunctional and functional PanNEN. A high NLR has also been proven to be a risk factor of recurrence in nonfunctional PanNEN. In contrast, the NLR was statistically unrelated to RFS in functional PanNEN. One reason for this might be that we analyzed a relatively small number of the patients with hormonally functional PanNEN; only three patients in this subgroup had recurrence during the follow-up period.

Surveillance at shorter intervals might be required in patients with nonfunctional well-differentiated PanNEN with a high NLR and 2017 WHO G2/G3 classification to detect recurrence earlier after surgery. Furthermore, almost all well-differentiated PanNENs express somatostatin receptors; hence, somatostatin receptor scintigraphy should be considered in PanNEN patients with a high NLR to help identify distant metastases that could be missed by computed tomography or positron emission tomography before and after surgery [38–40]. Well-differentiated PanNEN with the risk factors described above may receive clinical benefits by adjuvant treatments such as somatostatin analogs after surgery. However, there is no current evidence or clinical indication for adjuvant therapy, and further studies that focus on these high-risk groups are required.

This study had some limitations. First, it was a retrospective review of a single, high-volume institution in the field of pancreatic tumors. Several studies reported the NLR cutoff values as a prognostic factor, but the cutoff values were different across these studies due to the variation in disease stage or heterogeneity of the patient population. Therefore, we need to perform the external validation study in a large population using a nationwide clinical database or multi-center trial to confirm our findings in the future. Second, although consecutive patients were enrolled, they were collected over a relatively long period, during which treatment strategies changed reasonably.

## Conclusion

In summary, the results of our present study clearly demonstrated that the NLR could serve as a useful preoperative marker of clinical recurrence risks after the surgery. It is considered a convenient screening tool for the host immune response and should be incorporated into preoperative workups in the clinical management of well-differentiated PanNEN patients.

## Supplementary Information

**Additional file 1: Fig. S1.** ROC curve for the NLR and tumor size in well-differentiated PanNENs. The ROC curve illustrated that NLR has an AUC of 0.664 (95% CI 0.464–0.864) and tumor size has an AUC of 0.801 (95% CI 0.675–0.945). Arrows indicate optimal cut-off values. **Fig. S2.** Recurrence-free-survival and overall survival for PanNENs stratified by the 2017 WHO classification. Recurrence-free-survival and overall survival of neuroendocrine carcinoma (NEC) were significantly shorter than those of well-differentiated PanNENs (NET G1-3). **Fig. S3.** Distribution of NLR in PanNENs stratified by the 2017 WHO classification. The value of NLR in patients with NEC was significantly higher than that in patients with well-differentiated PanNEN.

## Data Availability

The datasets used and analyzed during this study are available from the corresponding author upon reasonable request.

## Abbreviations

PanNEN: Pancreatic neuroendocrine neoplasm
NLR: Neutrophil–lymphocyte
MLR: Monocyte–lymphocyte ratio
PLR: Platelet–lymphocyte
PWR: Platelet–white blood cell ratio
RFS: Recurrence-free-survival
OS: Overall survival
