# Supplementary figures and images for: Increased neutrophil–lymphocyte ratio predicts recurrence in patients with well-differentiated pancreatic neuroendocrine neoplasm based on the 2017 World Health Organization classification
Source: BMC Surg. 2021 Mar 31;21:176. doi: 10.1186/s12893-021-01178-3 (PMC8011407; doi:10.1186/s12893-021-01178-3)

## Slide 1
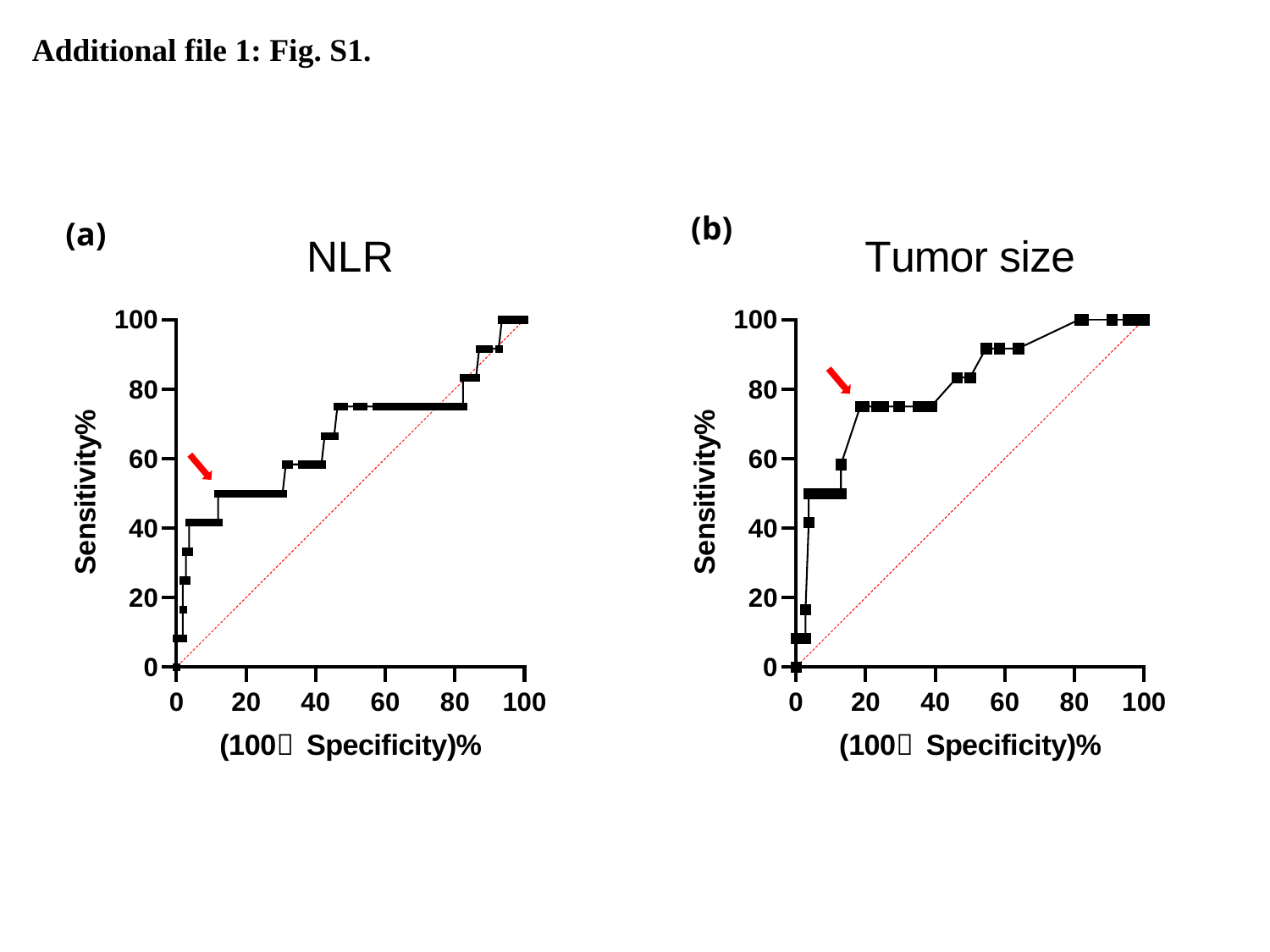

Additional file 1: Fig. S1.
(b)
(a)

## Slide 2
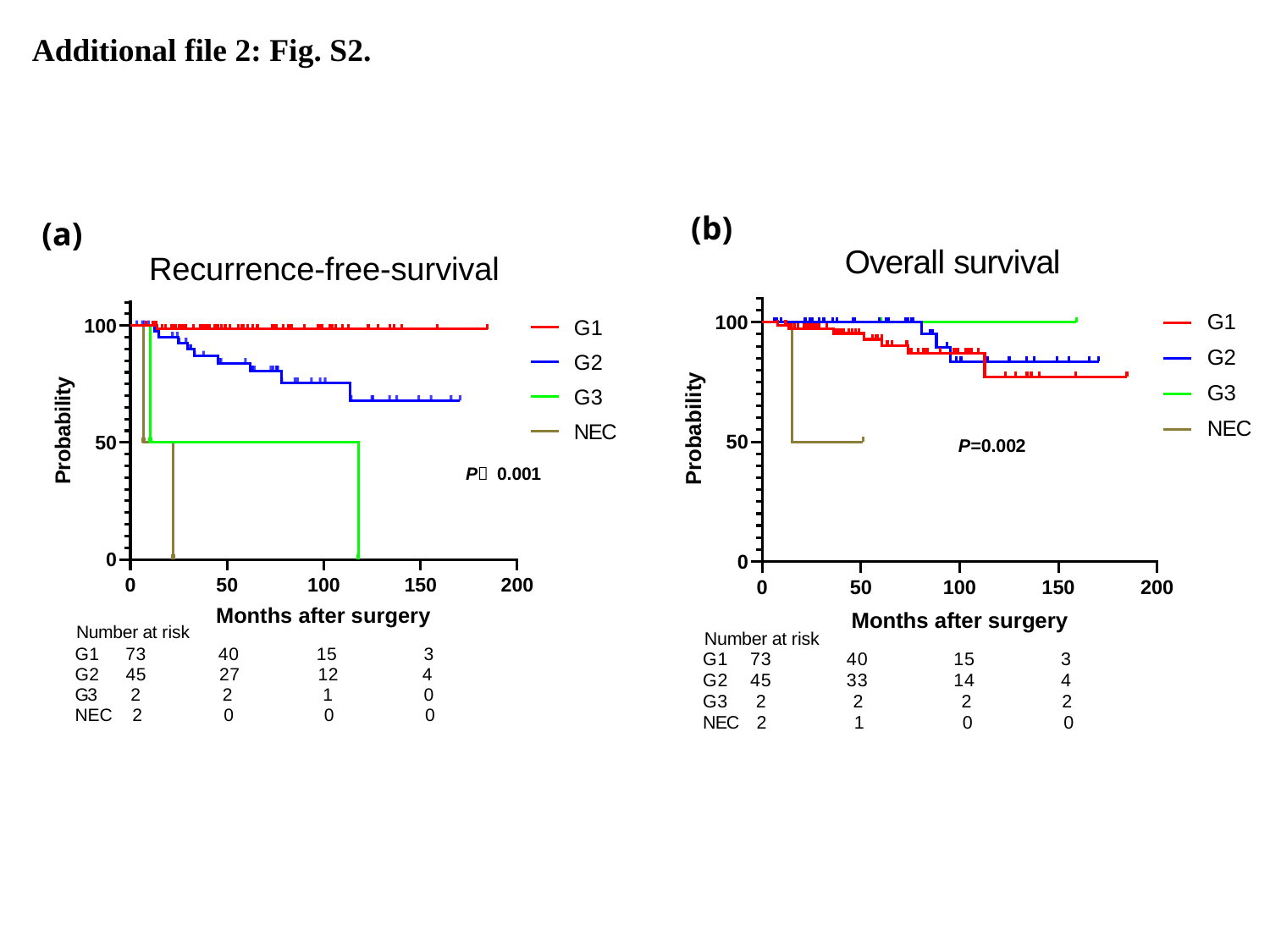

Additional file 2: Fig. S2.
(b)
(a)

## Slide 3
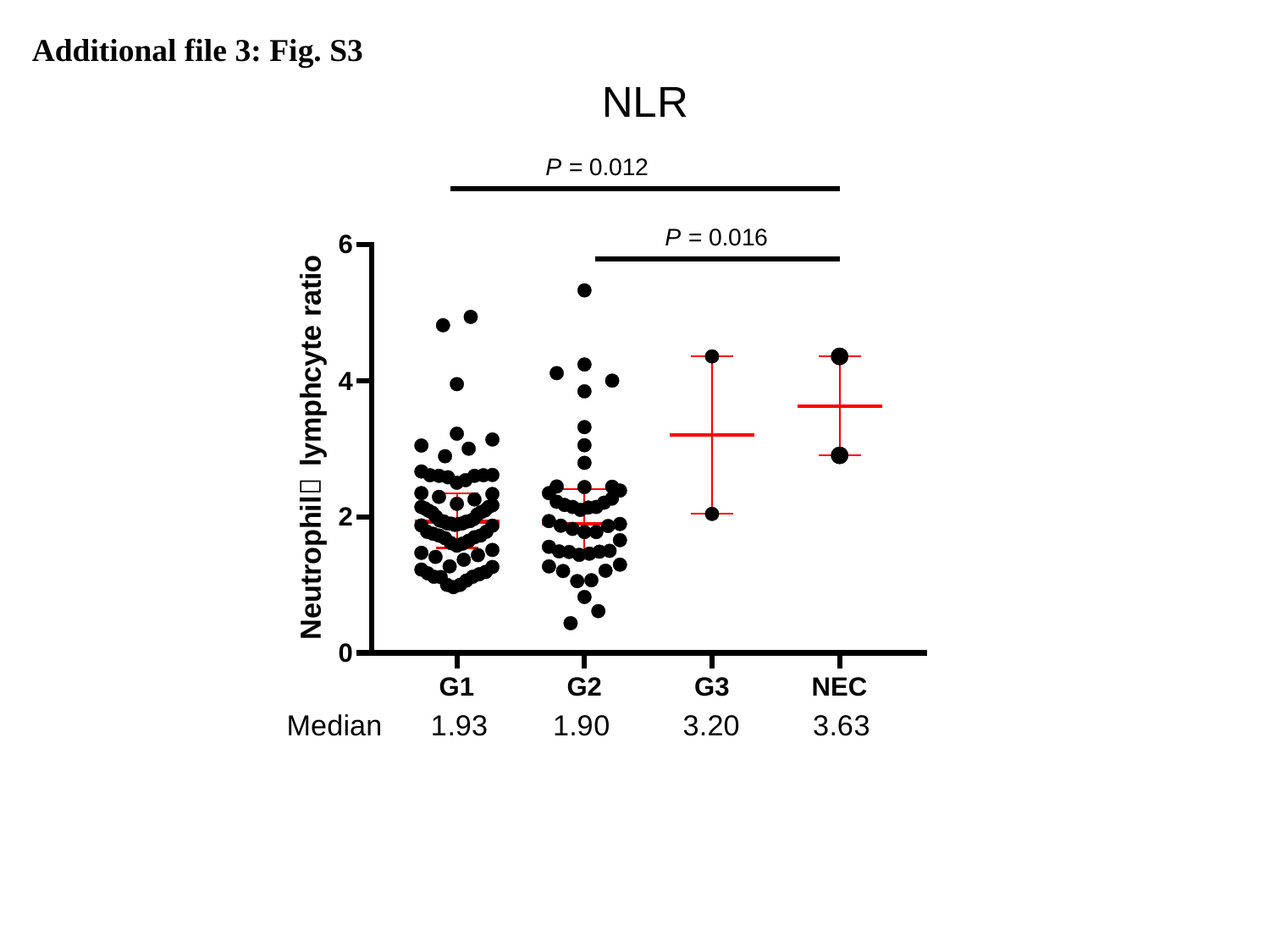

Additional file 3: Fig. S3

Supplement: Supplementary file 1 — Additional file 1: Fig. S1. ROC curve for the NLR and tumor size in well-differentiated PanNENs. The ROC curve illustrated that NLR has an AUC of 0.664 (95% CI 0.464–0.864) and tumor size has an AUC of 0.801 (95% CI 0.675–0.945). Arrows indicate optimal cut-off values. Fig. S2. Recurrence-free-survival and overall survival for PanNENs stratified by the 2017 WHO classification. Recurrence-free-survival and overall survival of neuroendocrine carcinoma (NEC) were significantly shorter than those of well-differentiated PanNENs (NET G1-3). Fig. S3. Distribution of NLR in PanNENs stratified by the 2017 WHO classification. The value of NLR in patients with NEC was significantly higher than that in patients with well-differentiated PanNEN. [file 12893_2021_1178_MOESM1_ESM.pptx]
